# Harnessing omics to refine taxonomic classification in the malaria vector *Anopheles gambiae species* complex: a review

**DOI:** 10.1186/s12936-026-06041-5

**Published:** 2026-07-30

**Authors:** Trizah K. Milugo, Pilate Kwi, Mamadou O. Ndiath, Benoit S. Assogba, Alfred Amambua-Ngwa

**Affiliations:** 1 Medical Research Council Unit The Gambia at LSHTM, Banjul, The Gambia; 2 Wellcome Sanger Institute, Hinxton, Cambridge, UK

**Keywords:** *An. gambiae* complex, Omics, Taxonomic identification, Malaria vectors

## Abstract

The malaria vector *Anopheles gambiae* s.l. (Diptera: Culicidae) is predominant in sub-Saharan Africa, where it plays a crucial role in transmitting malaria parasites to humans. Hitherto considered a single species, advances in cytogenetics and genomics have unveiled members of the complex as separate species. Whilst these species are morphologically indistinguishable, they are genetically distinct exhibiting significant differences in their ecological interactions and behavior. For example, they display considerable variation in their response to vector control measures and capacity to host malaria parasite, contributing differentially to malaria transmission. Consequently, precise taxonomic identification might help guide control programs against them. However, current approaches used in *An. gambiae* taxonomic systematics differ in accuracy and sensitivity, making it difficult to correctly delimit species boundaries. In this review, we discuss available taxonomic approaches utilized in *An. gambiae* complex whilst providing a framework for future taxonomic efforts. We also examine the process of naming a new species within the complex and detail the complexities of existing nomenclature approaches, as well as the prospective challenges they present, while advocating for an alternative approach. We conclude by providing a perspective on the use of a robust integrated approach in classifying and naming species of the *An. gambiae* complex.

## Background

The concept of identifying and organizing species into groups to understand their phylogenetic and evolutionary relationships, is a centuries-old practice that is widespread across many disciplines [1]. In the context of mosquitoes, where some species are incriminated as vectors of human malaria, taxonomy has proved essential, having wide-range applications. First, taxonomic knowledge is critical to monitoring existing mosquito species and surveying for new or invasive ones [2]. Second, precise species identification is required to distinguish disease vectors from non-vectors which is central to the implementation of targeted control [3, 4]. Third, proper species identification is needed to guide the application of insecticides as resistance mechanisms may vary across mosquito species or populations [5, 6]. Moreover, accurate taxonomy can contribute to the understanding of malaria transmission dynamics as host preferences [7, 8] susceptibility to parasites [9], and resting habitats [10, 11] may vary among vectors. Another application in view is in gene flow dependent genetically-modified vector control technologies (i.e. gene-drive) where accurate taxonomy may inform species-specific dispersal of gene-of-interest across mosquito populations [12].

The landscape of taxonomic systematics encompasses several approaches of varying benefits and limitations (Table 1). In recent years, the architecture of this landscape has undergone considerable transformation driven largely by the availability and increased accessibility of genome sequencing technologies and advances in computational resources. These advances have increased taxonomic accuracy and speed but in concert, made species delimitation quite challenging for those without solid genomic background especially in reference to accurate assignment of hybrid/intermediates or highly divergent lineages. This perhaps explain the designation of “unknown” Anopheline spp. in some entomological field survey [13–15].

**Table 1:** Traditional techniques employed in *Anopheles gambiae* complex taxonomy

Techniques	Application	Limitations	References
Morphology	Initial identification of species	Possible loss of diagnostic features [i.e. setae and scales] during collection or storage Expertise is drastically reducing Not accurate for cryptic species as speciation may occur without change in morphological features Can be subjective in assigning species	[16, 17]
Molecular methods DNA barcoding & Sequencing	Suitable for delineating species complex Suitable for immature stages Can work on samples stored inadequately Rapid identification & objective Reveal additional insights into evolutionary relationships	Accuracy can be compromised by hybridization and introgression Use of single locus underestimate species diversity while WGS is expensive Incomplete lineage sorting leading to inconclusive identification	[16–21]
Cytogenetics and Karyotyping	Are species specific and use to identify cryptic species Can reveal evolutionary trends, and adaptation Can reveal reproductive isolation	Response to environmental factors such as pollution can limit accuracy of species delimitation Limited resolution due to complexity of the genome Subjectivity in data interpretation	[18, 22, 23]
Biochemical approaches i.e. Isoenzymes	Are species specific and use to identify cryptic species Can reveal evolutionary trends	Limited resolution for closely related species Expertise is drastically reducing	[24]
Cuticular hydrocarbons	Might reveal cryptic species Important for chemotaxonomy (classification based on chemical traits) Can provide insights into evolutionary relationships	Prone to interference by environmental factors (Diet, temperature, humidity) and biological factors (Age, sex) and processes (mating) Standard protocols are yet to be established	[25–27]
Matrix-assisted laser desorption/ionization time-of-flight mass spectrometry (MALDI-TOF–MS)	Discriminate sibling species	Standard protocols are yet to be established Technically demanding	[19, 28, 29]

In the ensuing sections, we review approaches commonly used to elucidate the taxonomy of genetically distinct species belonging to *An. gambiae* complex. We also present a balanced discussion on recent advances in genomics and its application in taxonomy, while providing insight into alternative approaches for species nomenclature. Besides, we discuss explicitly the necessity of integrating other omics approaches in advancing *An. gambiae* taxonomy, especially for emerging species still undergoing reproductive isolation (hybrids).

### Taxonomic approaches to *An. gambiae* species description and systematics

Use of morphological features is the most applied criterion for assigning field collected mosquitoes into pre-existing taxa [30, 31]. However, for species complexes, accurate morphological identification is difficult as speciation often proceed without any noticeable morphological changes thus complicating visual delimitation of species boundaries [32, 33]. As such, a combination of methods is generally deemed appropriate for accurate identification and classification of cryptic species complexes. Preferably, taxonomic keys based on external morphological features found on the mosquito head, thorax, abdomen, legs and wings, are used as a first step to classify species [34, 35]. Thereafter, subsequent analysis is achieved via additional tools such as chromosome analysis (cytogenetic and karyotyping) and DNA markers (Table 1). Other complementary methods include cross-mating and allozyme analysis, though they are generally time consuming, they make molecular approaches more attractive especially when cryptic species are involved [18, 31, 36]. However, morphological identification remains widely preferred for its low cost and simplicity, allowing specimen identification in the field [18].

**Fig. 1:**
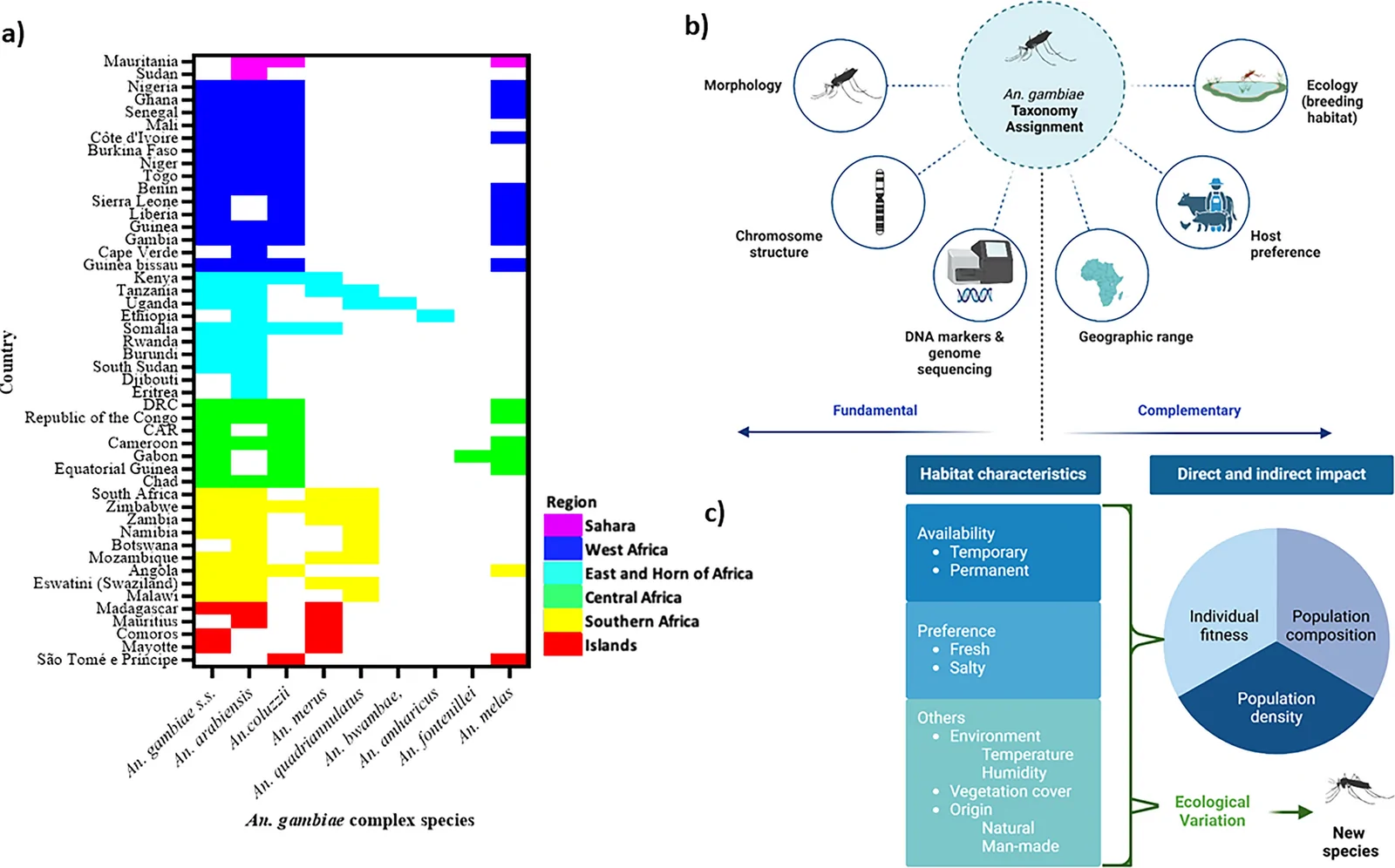
**a** Geographical distribution of *An. gambiae* species complex in SSA by country. Color denotes region, Red (Island nations), yellow (South Africa), Green (Central Africa), Aqua (East Africa), blue (west Africa), Pink (North Africa). DRC (Democratic republic of Congo), CAR (Central Africa republic) [39–52]. **b** Taxonomic techniques and other necessary prerequisite for accurate classification of species into *An. gambiae* complex. **c** Habitat characteristic and preference can influence population density and ecological speciation

The sibling species in *An. gambiae* s.l complex (Diptera: Culicidae) include *Anopheles arabiensis*, *Anopheles coluzzii*, *An. gambiae* s.s, *Anopheles melas*, *Anopheles merus*, *An. quadriannulatus*, *Anopheles bwambae*, *Anopheles amharicus* and the recently described *Anopheles fontenillei* [2, 37]. Of these, *An. coluzzii, An. gambiae* s.s and *An. arabiensis* are the dominant malaria vectors across sub-Saharan Africa (SSA) [38] (Fig. 1a). The assignment of members within this complex depends on both morphological and molecular techniques as described earlier, but also takes into account additional criteria like habitat preference, geographic range, and host preference (Fig. 1b). This multiple lines of evidence helps to robustly unravel cryptic species without overestimating species number.

***Habitat preference:*** Members of the *An. gambiae* complex are ecologically differentiated at larval stages exhibiting variations in habitat preference [53–55]. For example, although *An. gambiae* s.s and *An. coluzzii* occur in sympatry and prefer freshwater breeding sites [56], the latter exhibits a tolerance to salinity allowing it to survive in polluted water [57]. Likewise, the recently identified *An. fontenillei*, while closely related to *An. bwambae* which resides in mineral-rich hot springs, prefers freshwater as *An. gambiae* s.s [2]. Other salt breeders in the complex are *An. melas* and *An. merus* whilst *An. arabiensis*, demonstrates more flexibility, thriving in freshwater while tolerating moderate salinity [58]. Generally, breeding habitat selection is facilitated by the ability of gravid females to detect specific cues related to water properties and volatile chemicals at the breeding site [59]. This perhaps explains the rarity of shared breeding sites among some sibling species even when they occur in sympatry in a given geographic space [53]. This variation in breeding site choices is also influenced by other parameters such as habitat availability (temporary or permanent) and origin (natural or man-made) (Fig. 1c). For example, whilst *An. gambiae* and *An. coluzzii* prefer natural water bodies [53], others like *An. arabiensis* have a preference for man-made and temporary habitats [53, 58, 60]. Habitat characteristics may influence larval survival, pupation duration and productivity, all of which directly impact individual fitness and indirectly influence population growth and density (Fig. 1c) [61, 62]. Besides, it is posited that contrasting preferences of larvae to breeding habitats can contribute to ecological speciation, a concept that has been demonstrated for *An. coluzzii* in both field [60] and laboratory experiments [63–65].

**Geographic range:** Distribution of member species of *An. gambiae* in Africa occur over a broad geographic range encompassing divergent environments and ecologies. Of the nine taxa, *An. gambiae* s.s and *An. arabiensis* exhibits vast ecological and geographical distribution, ranging from west to east Africa, often occurring in sympatry (Fig. 1a). This widespread distribution across the continent may be attributable to their ecological adaptability and availability of suitable breeding sites [66, 67]. Likewise, *An. coluzzii* also displays a wide spread geographical distribution. Although found commonly in West and Central Africa [21, 38], it has recently been detected in Somalia and Kenya [57]. Changes in climate, making previously inaccessible areas habitable, could be the main ecological driver behind *An. coluzzii* dispersal to East Africa [68]. The other species are more geographically restricted with *An. melas* and *An. merus* being found in coastal areas of West and East Africa, respectively (Fig. 1a). The remaining two, *An. bwambae* and *An. amharicus*, are also limited in their distribution to east Africa (Fig. 1a) [52, 69].

**Host preference:** The members of *An. gambiae* species complex also display broad host range with differing preferences [38, 70, 71]. The most anthropophilic (feed on humans) species of the complex and thus key players in human malaria transmission are *An. coluzzii* and *An. gambiae* s.s [7, 72, 73]. Other members are zoophilic (feed on animals): (*An. quadriannulatus, An. melas, An. merus, An. bwambae* and *An. amharicus*) and play a minor role in human malaria transmission [48, 74–77]. The exception is *An. arabiensis*, which despite being zoophilic, displays opportunistic human feeding behavior, facilitating transmission of human malaria outdoors [38, 45].

### Species delimitation using genomics: advancement and challenges

**Fig. 2:**
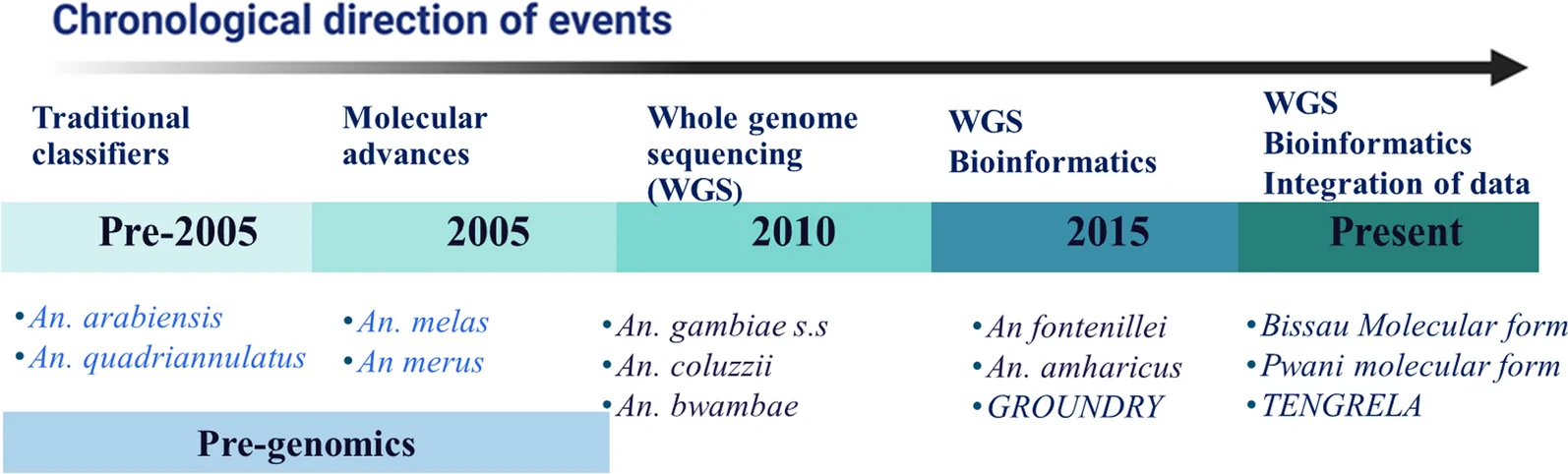
Schematic representation summary of *An. gambiae* species identification in pre and present era of genomics

Recent advances in genomics and bioinformatics have made it possible to genetically distinguish members of *An. gambiae* species complex with increased accuracy. Moreover, genomic analysis approaches are projected to uncover more members of the complex beyond the nine currently described. This is exemplified by the recent addition of two novel taxa in the complex termed Bissau molecular form detected in west Africa [78] and Pwani molecular form reported in east Africa [79]. Bissau molecular form for example is genetically distinct from its sibling species (*An. gambiae* and *An. coluzzii*) and could have evolved simultaneously during ancestral species split [78]. Other novel taxa in the complex are GROUNDRY and TENGRELA, also close relatives of *An. coluzzii*, and were first reported in west Africa [80, 81]. Their role in malaria transmission however still remain uncertain. Also undefined are the ecological drivers influencing their geographical distribution. Nonetheless, these recent findings allude to species richness within the Gambiae complex, emphasizing the need to explore additional ecologies across Africa for novel species. Furthermore, comparative analysis of genomic timelines from 2010 (overlapping with whole genome sequencing) vs species taxonomic assignment points to the potential of genomics analysis in unravelling novel members of *An. gambiae* complex (Fig. 2).

Another recent advancement in genomics is recognized in the assignment of *An. coluzzii* as a distinct species in the Gambiae complex [37]. Though identified earlier using other methods, genome sequencing enabled its elevation to species level following clarification of existing ambiguities in the speciation continuum of *An. gambiae* chromosomal forms [82, 83]. Notably, five chromosomal forms have been previously described (Mopti, Bamako, Bissau, Forest and Savannah), but only two, Mopti and Savannah, have their taxonomic status resolved [84]. The M (now *An. coluzzii*) form is associated with Mopti while the S form (also *An. gambiae*) is associated with Savannah and in some instances Forest or Bamako [20, 23, 84–86].

*New advances in genomics*: At present, there is an on-going inventory of *An. gambiae* complex genomes on different genomic epidemiology initiatives. The most extensive initiative enabling delimitation of species boundaries is the MalariaGEN’s *An. gambiae* 1000 genomes project, which has a genome sequence database of over 30,000 mosquitoes species from multiple African countries [87]. Another hallmark of MalariaGEN includes open source bioinformatics applications that can be exploited to simultaneously address multiple questions, such as genetic diversity, population structure [88], insecticide resistance [89], vector competency [90], evolution and phylogenetic relationships [91] in different sibling species of *Anopheles gambiae* mosquitoes.

Genome sequencing typically generates large sets of polymorphism data requiring algorithms and data processing tools for accurate and rapid speciation. Consequently, the genome sequencing pipeline involves application of various data analysis tools which occasionally may result in inconsistent interpretation [92]. Inconsistencies could stem from differences in reference genomes used and/or parameter settings. As such, harmonizing data analysis tools and additional species specific reference genomes could enhance robustness in the identification and classification of species. More specifically, it will help clarify relationships in closely related taxa, deconvoluting the complexity that underlies mosquito population diversity and response to insecticide-based vector control measures [93, 94].

*Other critical considerations*: To effectively use genomics to advance *An. gambiae* complex taxonomy, the following factors should be taken into consideration i) improved accessibility and reduced cost of genome sequencing especially to researchers in low income countries where financial resources are limited, ii) qualification of various taxonomic targets (multi-loci markers) with sufficient discriminatory power; iii) leveraging various mosquito life cycles to enhance taxonomic accuracy; and iv) standardizing genomic analysis tools and guidelines to reduce subjectivity in data interpretation.

### Nomenclature of *An. gambiae* s.l: challenges and prospects

Species nomenclature follows identification and classification and is important for effective scientific and public health communication. For malaria vectors, naming system should aim at standardization and scalability, allowing renaming of newly identified mosquito species whenever necessary. As genome sequencing increases resolution, nomenclature changes are likely to occur as the large amount of genetic data-driven molecular systematics outpace previous consensus among scientists on species naming. Unfortunately, there are currently no universally aggreed nomenclature rules for newly identified *Anopheles* species from genomic data.

Ideally, naming a new species requires finding a novel exclusive binomial nomenclature. This is quite challenging, and as such, it is not unusual to find mosquito species named after individuals [95], locations or vegetation type. For example, in *An. gambiae* s.s the naming of chromosomal forms (Mopti, Bamako, Bissau, Forest and Savannah) were based on geographical areas or habitat type to describe adaptation of this species to environmental conditions [23, 86]. This system of naming may however be misleading to some policy makers, as they may assume that the species are restricted to specific geographical areas. This misconception can result in some mosquito species being erroneously overlooked by routine surveillance and malaria elimination efforts. On the other hand, there is also a challenge with naming species after scientists as this might only find meaning within the specific scientific community. Preferably, species nomenclature should be descriptive, highlighting features with the potential to guide control efforts such as behavior, physical trait, ecological role or role in disease transmission.

Here we propose the use of specific genome-based identifiers linked to the genus, for species that are yet to be fully characterized. Since genomics data are processed electronically, generating a unique identifier (preferably a code) for a newly sequenced genome is feasible and essential. The MalariaGen initiative has made attempts towards this direction by coding newly identified taxa in the *An. gambiae* complex as gcx-1 to gcx-4 [78, 79, 87]. Such an approach will streamline species naming and support future taxonomic applications, including species renaming and retrieval of names for further characterizations. Moreover, having a stable, organized and consistent naming convention will help to minimize taxonomic uncertainties as vector species continue to diversify, due to climate change and other environmental pressures. Notably, the proposed naming concept aims only to provide insights, and is not conclusive, needing further discussion among vector ecologist.

### Future directions

**Fig. 3:**
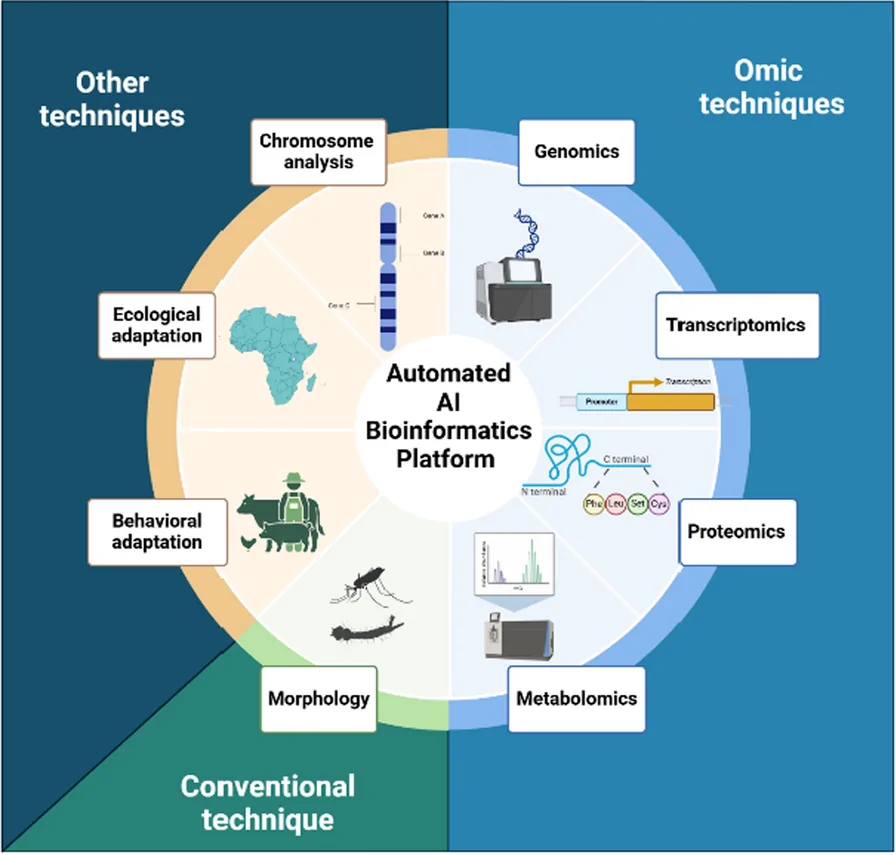
An illustration depicting an integrative taxonomy approach combining multiple lines of evidence beyond the traditional approaches

As outlined in the current review, available taxonomic techniques present several intriguing challenges including the potential to underestimate species diversity for classifiers based on morphology, and for molecular methods, the inability to make decision on species boundaries due to admixture and complexity of genomic data. In nature, speciation is not instantaneous but rather a gradual evolutionary process involving multiple events following reproductive isolation, following which adaptive introgression, gene flow and lineage sorting impose challenges to taxonomic assignment [75]. To generate classification that is useful for the malaria community, an integrative taxonomy is deemed necessary. This will enhance species assignment with greater reliability and reproducibility while reducing subjectivity. Already, some recent works have adopted this approach by integrating genetic sequences (molecular) and images (morphological data), which together with Bioinformatics and deep learning methods (Convolutional Neural Network (CNN)) increases the robustness in delineating species [96]. Indeed, the use of machine learning was reported to have high accuracy in classifying mosquito species to genus and species level [97]. However, what remains unresolved is a platform that can combine genomic datasets with morphological data to enable species delimitation with utmost accuracy and speed. Artificial intelligence (AI), both integrative and rapid, has this potential and offers the added advantage of being less subjective (Fig. 3). Present AI tools have advanced to the extent that they can simulate human reasoning, learn new information and make predictions. Thus combining generative AI, machine and deep learning with human expertise can transform and accelerate taxonomic systematic.

*Non-genomics omics*: Usually the initial motivator for discovery of sibling species is association with parasite infection. Yet simple genetic locus may show that they do not conform to known species in GenBank or sometimes identification may be complicated by lack of reference sequence in public databases. Besides genome variation data, other omics approaches such as metabolomics, transcriptomic, and proteomics may also be exploited to enable an all-inclusive approach to species identification and classification. The combined multi-omics system would be more appropriate in circumstances where sibling species display salient behavioral and ecological traits but are genetically similar, perhaps due to recent divergence. Already, metabolomics has been deployed successfully in identifying members of the genus Aspergilus [98, 99] and Ananas at the species level [100]. But in mosquitoes, metabolomics profiling as well as transcriptomic and proteomics for delimitation of species boundaries is unexplored.

Proteomics also hold a lot of promise in species delimitation, but is currently underutilized in comparison to genomics or transcriptomic [101]. At present, its application in taxonomy is more exploited in microbiology [102, 103] whilst in Anopheles mosquitoes, it's mainly aimed at understanding cellular functions [101, 104]. Perhaps, the availability of high-resolution mass spectrometers and advanced bioinformatics tools for processing proteomics data (i.e. amino acid sequences alignment and clustering) may enhance its uptake in mosquito taxonomy.

Transcriptomic, also functional genomics, is another omics that can deepen our understanding of mosquito taxonomy. Already, transcriptomic sequencing (RNA seq, mRNA seq and gene expression profiling) is available on various sequencing platforms. Often, this omic is used to understand gene expression profiles following mosquito exposure to various factors of biological or environmental origin, including insecticides and other xenobiotics. So far, positive records exist of its use in understanding genetic diversity in Culex mosquito [105], but its application in the taxonomy of Anopheles mosquitoes remains unexplored.

Due to the potential huge amount of data and cost related challenges, adopting multiomic approach for every species group might prove challenging, nonetheless it can be prioritized in cases where ecological divergence is anticipated. As indicated earlier, an integrative approach that unifies and standardizes omics tools with all the tools used in taxonomy, including machine learning, will be necessary to have consistent results (Fig. 3). Moreover, it will facilitate the establishment of an online pipeline for querying taxonomy that is not only comprehensive but accurate and interactive.

## Conclusion

Taxonomy has application in multiple disciplines including evolutionary genetics, population genetics and epidemiology. This review highlighted the major methods used in *An. gambiae* complex taxonomy, their strength and shortcoming. The specific concepts discussed herein include taxonomic techniques, advances in genomics and bioinformatics, and integration approach to taxonomy. Specifically, we highlighted how genome sequencing can contribute to refining classification of species in *An. gambiae* complex, and advocated for its use in taxonomy without suggesting it as the gold standard. Instead we proposed a comprehensive framework that incorporates other existing techniques including bioinformatics algorithms and AI. Most importantly, we argued for a standardized integrated pipeline for achieving accurate taxonomy. We also addressed some issues in current species nomenclature and offered suggestion for future naming practices that are more pragmatic and informative, meeting the needs of both scientists and policy makers. Noteworthy, our discussion centered on *An. gambiae* complex but many of the concepts may be applicable to other mosquito species complexes or disease vectors. Presently, it is widely accepted that effective and targeted control of disease vectors will require an in-depth knowledge of vector genomics, biology, ecology as well as accurate taxonomy. In tropical Africa for example, malaria transmission is often heterogeneous enabled by species belonging to different Anopheles complexes and without a clear understanding of their systematics, targeted control might prove difficult. Overall, we hope that the ideas presented herein will spark greater interest and discussions among taxonomist and vector ecologist.

## Data Availability

No datasets were generated or analysed during the current study.

## Abbreviations

AI: Artificial intelligence
CAR: Central Africa republic
CNN: Convolutional Neural Network
DRC: Democratic republic of Congo
MALDI-TOF–MS: Matrix-assisted laser desorption/ionization time-of-flight mass spectrometry
SSA: Sub-Saharan Africa
WGS: Whole genome sequencing
